# A dual-binding magnetic immunoassay to predict spontaneous preterm birth

**DOI:** 10.3389/fbioe.2023.1256267

**Published:** 2023-09-13

**Authors:** Michael Sveiven, Andrew Gassman, Joshua Rosenberg, Matthew Chan, Jay Boniface, Anthony J. O’Donoghue, Louise C. Laurent, Drew A. Hall

**Affiliations:** ^1^ Department of Bioengineering, University of California, San Diego, La Jolla, CA, United States; ^2^ Sera Prognostics, Inc., Salt Lake City, UT, United States; ^3^ Department of Electrical and Computer Engineering, University of California, San Diego, La Jolla, CA, United States; ^4^ Skaggs School of Pharmacy and Pharmaceutical Sciences, University of California, San Diego, La Jolla, CA, United States; ^5^ Department of Obstetrics, Gynecology, and Reproductive Sciences, University of California, San Diego, La Jolla, CA, United States

**Keywords:** preterm birth, point-of-care testing, sex hormone-binding globulin, SHBG, insulin-like growth factor-binding protein 4, IBP4, giant magnetoresistive sensor, GMR

## Abstract

Complications posed by preterm birth (delivery before 37 weeks of pregnancy) are a leading cause of newborn morbidity and mortality. The previous discovery and validation of an algorithm that includes maternal serum protein biomarkers, sex hormone-binding globulin (SHBG), and insulin-like growth factor-binding protein 4 (IBP4), with clinical factors to predict preterm birth represents an opportunity for the development of a widely accessible point-of-care assay to guide clinical management. Toward this end, we developed SHBG and IBP4 quantification assays for maternal serum using giant magnetoresistive (GMR) sensors and a self-normalizing dual-binding magnetic immunoassay. The assays have a picomolar limit of detections (LOD) with a relatively broad dynamic range that covers the physiological level of the analytes as they change throughout gestation. Measurement of serum from pregnant donors using the GMR assays was highly concordant with those obtained using a clinical mass spectrometry (MS)-based assay for the same protein markers. The MS assay requires capitally intense equipment and highly trained operators with a few days turnaround time, whereas the GMR assays can be performed in minutes on small, inexpensive instruments with minimal personnel training and microfluidic automation. The potential for high sensitivity, accuracy, and speed of the GMR assays, along with low equipment and personnel requirements, make them good candidates for developing point-of-care tests. Rapid turnaround risk assessment for preterm birth would enable patient testing and counseling at the same clinic visit, thereby increasing the timeliness of recommended interventions.

## Introduction

The average length of human gestation is approximately 40 weeks, and birth is considered preterm before 37 weeks. Globally, preterm births affect 15 million infants annually and are strongly associated with adverse postnatal outcomes, such as developmental and intellectual disabilities, including cerebral palsy, attention deficit hyperactivity disorder, depression, and anxiety (Liu et al., 2012; Luu et al., 2017). Preterm birth is also associated with long-term pulmonary complications, such as asthma and bronchopulmonary dysplasia. In addition, preterm birth increases the risk of diabetes, dental problems, hearing loss, and infections (Saigal and Doyle, 2008; Parkinson et al., 2013; Li et al., 2014; Boyle et al., 2017). The UN Inter-agency Group for Child Mortality Estimation reported in 2019 that preterm births caused 35% of global neonatal deaths. About 1 in 10 babies in the United States are born preterm, at an estimated $25 billion cost to the healthcare system annually (Waitzman et al., 2021). The earlier the birth, the more serious the health and financial consequences are, indicating that prolonging pregnancies would yield important gains (Petrou et al., 2019; Waitzman et al., 2021).

Currently, the treatment of preterm labor is largely reactive, with tocolytics, antenatal corticosteroids, and magnesium sulfate being offered to pregnant individuals with signs or symptoms of preterm labor (Haghighi et al., 2017; Conde-Agudelo et al., 2018). Antenatal corticosteroids, such as betamethasone and dexamethasone, are administered to pregnant women at high risk of delivery within the subsequent 2 weeks to accelerate infant lung development and prevent perinatal complications, such as respiratory distress syndrome, intraventricular hemorrhage, and necrotizing enterocolitis (Autran et al., 2018; Conde-Agudelo et al., 2018). Magnesium sulfate administered shortly before early preterm birth (prior to 34 weeks gestational age) decreases the risk of cerebral palsy (Costantine and Weiner, 2009; Doyle et al., 2009). Tocolytics such as beta-adrenergic receptor agonists, calcium channel blockers, and nonsteroidal anti-inflammatory drugs can delay labor—but only for a few days; fortunately, this delay is often sufficient for the administration of antenatal corticosteroids and magnesium sulfate (Haas et al., 2012; Flenady et al., 2014; Wilson et al., 2022).

For pregnant individuals with an elevated risk of preterm birth due to a history of preterm birth or an ultrasound finding of a short cervix, weekly intramuscular treatments with 17-hydroxyprogesterone or daily treatments with vaginal progesterone are initiated between 16–24 weeks gestational age (GA). These treatments have been shown to prevent preterm birth, although their efficacy across different categories of at-risk individuals has been debated (Meis et al., 2003; Fonseca et al., 2007; Akinwunmi and Ming, 2022; Boelig et al., 2022; Conde-Agudelo and Romero, 2022; Lin and Nie, 2022; Nelson et al., 2022). Alternatively, care management, which encompasses coordinated care aimed at providing a more comprehensive and supportive environment, may improve the environmental, behavioral, social, and psychological factors contributing to the risk of preterm birth (Garite and Manuck, 2022). Unfortunately, nearly 70% of spontaneous preterm births (sPTBs) occur in first pregnancies or in pregnancies where the mothers have no history of preterm birth, and for these individuals, progesterone or monitoring of cervical length is not offered (Goldenberg et al., 2008). Thus, accurate and feasible risk assessment for sPTB has the potential to enable personalized clinical management with improved outcomes.

**FIGURE 1:**
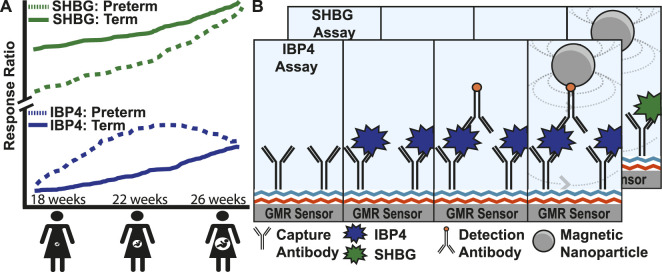
Magnetic immunoassay to identify pregnant women at high risk of preterm birth. **(A)** Illustration showing the increase of two proteins, SHBG and IBP4, in serum as pregnancy progresses. Women at high risk of preterm birth have abnormal levels of SHBG and IBP4 at different stages in the pregnancy where a proteomic score can be derived from the ratio of these proteins. **(B)** Magnetic immunoassay to quantify SHBG and IBP4 using a capture antibody bound to the sensor surface and a detection antibody that recruits magnetic nanoparticles (MNPs) close to the surface. The recruited MNPs perturb the local magnetic field, which is read out using the underlying giant mangetoresistive sensors. The IBP4 and SHBG immunoassays differ only by the antibody and analyte used, and therefore, the individual steps of the SHBG assay have been hidden to avoid redundancy in the image.

Diagnostic tools for monitoring pregnancy today are broadly classified into imaging and biomolecular tests. Serial transvaginal ultrasound measurement of cervical length is commonly used in pregnancies at high risk for sPTB, and a mid-trimester ultrasound screening of cervical length either transabdominally or transvaginally is routinely performed (Orzechowski et al., 2016; Booker et al., 2021). Serum biomarkers are used to evaluate ectopic and other nonviable pregnancies in the first trimester and to perform screening for open neural tube defects and fetal aneuploidy (PAPP-A, bHCG, AFP, Inhibin-A, and estriol) (Brock et al., 1975; Biggio et al., 2004; Xu et al., 2020; Betz and Fane, 2022). Fetal fibronectin found in cervicovaginal fluid is associated with preterm birth (Lockwood et al., 1991) with tests performed on vaginal swab specimens from gravidas at high risk of preterm delivery between 24–36 weeks GA to assess the risk of delivery within the following 1–2 weeks (Peaceman et al., 1997; Swamy et al., 2005). However, the utility of fetal fibronectin is limited by its poor positive predictive value of 17%–30% and the lack of evidence of its utility in improving clinical outcomes (Swamy et al., 2005; Son and Miller, 2017). Recently, proteomics has been used to predict complex diseases and outcomes. Sex hormone-binding globulin (SHBG) and insulin-like growth factor-binding protein 4 (IBP4) were identified as biomarkers for mothers at risk of sPTB (Saade et al., 2016; Bradford et al., 2017). Both proteins increase throughout pregnancy, but the trajectories diverge in pregnancies destined for preterm vs. term delivery (Figure 1A) (Kearney et al., 2018). Throughout pregnancy, SHBG increases 5- to 10-fold in human serum (Anderson, 1974; O’Leary et al., 1991), and its function is to bind hormones (*e.g.*, testosterone and oestradiol) to quench their activities. Insulin-like growth factor (IGF) is a hormone that stimulates body growth and development by acting on metabolic organs, including the liver, bone, and skeletal muscle. IBP4 regulates IGF activity through binding. In addition, IBP4 was previously identified as a biomarker for fetal growth restriction (Qiu et al., 2012).

From mass spectrometry assays, the algorithmic determination of sPTB risk using the combination of IBP4/SHBG biomarker abundance and clinical factors was found to have a high predictive value (Saade et al., 2016; Burchard et al., 2021). In pregnancies that subsequently deliver preterm, serum abundance of IBP4 is higher than normal, and SHBG levels are lower than normal between 18 and 22 weeks. A proteomic score combining the IBP4 and SHBG response ratios (the mass spectrometry response to an unknown sample divided by the response of a calibrant) can predict sPTB (Burchard et al., 2021). Mass spectrometry-based quantitation of SHBG and IBP4 is ideal for biomarker discovery and has successfully been extended to the clinic (Kearney et al., 2018), but it is not currently suitable in a point-of-care setting. Immunoassays are better suited for the rapid turnaround time of clinical samples. Two common immunoassay formats are the enzyme-linked immunosorbent assay (ELISA) and electrochemiluminescence immunoassay (ECLIA) (Richter, 2004; Crowther, 2008). ELISA uses an enzyme to convert substrate into a colorimetric or fluorogenic product. ECLIA uses electrodes to induce a luminophore into an excited state where it will emit light. Both assay formats require plate readers to capture the signal change. Thus, these tests require maternal blood samples to be sent off for processing and analysis. Processing requires expensive, highly specialized equipment and technical expertise exclusive to advanced laboratories. The optimal GA window to use SHBG and IBP4 for sPTB risk assessment is in gestation weeks 18–20. Decreasing the turnaround time may allow patients and clinicians to act sooner with interventional strategies. For example, the recommended GA window for initiating progesterone to prevent preterm birth is 16–24 weeks, and other interventional strategies, such as case management (Garite and Manuck, 2022), may benefit from early initiation. Moreover, failure to appropriately follow up results occurs in 7%–62% of laboratory tests (Casalino et al., 2009; Norwitz and Caughey, 2011; Callen et al., 2012). Test effectiveness may be improved to the extent that test result generation in a point-of-care setting can be coupled with better follow-up.

Recently, there has been a dramatic shift toward decentralizing diagnostic tests, making health information rapidly available to a patient’s healthcare provider (Arshavsky-Graham and Segal, 2022; Chen J. H. K. et al., 2020; Gong et al., 2019; Kumar et al., 2020; Mahmoudi, de la Guardia, and Baradaran, 2020). This adoption was further accelerated by the COVID-19 pandemic, where at-home testing became common. Point-of-care testing (POCT) brings the power of centralized labs directly to the patient, permitting testing in a healthcare office, at work, or at home. POCT for pregnant women may enable obstetricians to intervene quickly if a mother is at high risk for preterm birth. However, many POCT assay formats today (*i.e.*, lateral flow immunoassays) only test a single analyte and are not quantitative, preventing their use for this type of bivariate assay. Other POCT formats include electrochemical and optical (Cao et al., 2020; Chen Y. T. et al., 2020; Zhang et al., 2020). Magnetic sensors are particularly attractive for this application as they can be arrayed for multiplex detection, are already miniaturized, and are highly sensitive (Osterfeld et al., 2008; Gaster et al., 2011; Krishna et al., 2016; Klein et al., 2019). Giant magnetoresistive (GMR) sensors are thin-film proximity-based magnetic sensors where the local magnetic field is transduced into resistance change through a quantum mechanical effect (Barnaś et al., 1990; Prinz, 1998; Wolf et al., 2001; Osterfeld et al., 2008). As biological samples (*e.g.*, urine, saliva, serum, etc.) lack a magnetic background, this readout format is agnostic to the sample matrix, which greatly simplifies the sample preparation, which is often just dilution (Gaster et al., 2009). This constellation of properties may make magnetic sensors ideal for monitoring pregnant women in a point-of-use setting as they progress throughout their pregnancies.

In this study, we developed an immunoassay to quantify SHBG and IBP4 in serum samples that is amenable to point-of-care testing, thereby allowing the quantification of selected biomarkers clinically relevant to sPTB. Figure 1B illustrates the two assays where capture antibodies are immobilized on the sensor surface. When the maternal serum sample is added, the target analytes (IBP4 and SHBG) bind to their respective capture antibodies. Biotinylated detection antibodies are added and bind to the antigen forming a sandwich assay. The addition of streptavidin-coated magnetic nanoparticles (MNPs) results in binding to the detection antibodies quantitatively readout using the underlying GMR sensors. The change in magnetic signal during the assay is proportional to the target biomarker’s concentration in the serum sample. The GMR immunoassays quantify SHBG and IBP4 with sufficient sensitivity and accuracy in blood serum with a high degree of correlation to the proteins measured from a centralized laboratory assay based on mass spectrometry. Capable of being run in a point-of-care setting with minimal training required, the assay could provide obstetricians with a powerful tool to predict spontaneous preterm births and intervene when necessary.

## Materials and methods

### Reagents

Poly (allylamine) solution (PAAM; #479144), poly (ethylene alt maleic anhydride) (PEMA; #188050), Tween-20 (#P9416), and human serum (#H6914) were purchased from Sigma-Aldrich. Bovine serum albumin (BSA; #37525) was procured from Thermo-Scientific. Streptavidin-coated magnetic nanoparticles (#130-048-101) were acquired from Miltenyi Biotec. Human/Primate IL-6 Antibody (#MAB206) was obtained from R&D Systems. Fine crystalline 2-(4-Morpholino)ethane Sulfonic Acid (MES) and Tris-buffered saline (TBS, 10×) pH 7.4 (#J60764.K2) were purchased from Fisher Scientific (#BP300-100). Lauryl Maltose Neopentyl Glycol (#NG310) was bought from Anatrace. Phosphate-Buffered Saline (10×) pH 7.4, RNase-free (#AM9625), Pierce Bovine Serum Albumin, Biotinylated (#29130), and EZ-Link Amine-PEG11-Biotin (#26136) were procured through Thermo Fisher. Magnetic polyvinyl alcohol beads (#CMG-216) were procured through Perkin Elmer. Mouse Anti-Mouse IgD (#BDB553509) was obtained from Thermo Fisher. Betaine, 5M solution, molecular biology grade, Ultrapure (#AAJ77507AB) was acquired from Fisher Scientific. 2,3-Butanediol (#MFCD00004523) was obtained from Sigma Aldrich. Ethylene Glycol (#E178-500) was ordered from Fisher Chemical. Sera Prognostics provided SHBG and IBP4 antigens, monoclonal capture and detection antibodies against IBP4 and SHBG (Supplementary Table S1), and serum samples pooled from multiple unidentified female donors.

### GMR sensor arrays

GMR spin-valve (SV) sensor arrays were purchased from MagArray, Inc. (#BZ0078). Each GMR SV sensor array has 80 sensors arranged in an 8 × 10 matrix where each sensor is 120 × 120 μm^2^ on a 280 µm pitch with a nominal resistance (*R*_0_) of 1464 *Ω* and a mean magnetoresistance (MR) ratio of 7.99% (Supplementary Figure S1). Each one of the 80 sensors can be independently addressed. A custom holder was fabricated from Teflon to create a 100 µL reaction well with an o-ring on top of the sensor array (Supplementary Figure S2).

### GMR reader

The measurement setup consists of a computer, a power amplifier, a Helmholtz coil, and custom readout electronics, as shown in Supplementary Figure S3A (Hall et al., 2010a). A double modulation readout scheme was used to reject 1/*f* noise from the sensors and electronics, and a temperature compensation technique was used to reduce the temperature drift (Hall et al., 2010b). The computer digitally adjusted the frequencies and amplitudes of sensor bias voltage and magnetic field through a National Instruments data acquisition card (PCIe-6351) and a LabVIEW graphical user interface. Specifically, the power amplifier controlled by the computer provides current to the Helmholtz coil, which creates a homogenous magnetic field (23—34 Oe_rms_ based on the sensor MR) for the sensor array. The readout electronics contain 8× transimpedance amplifiers to convert the currents to voltages that were quantized by the acquisition card. Time-multiplexing was applied to read the 8 × 10 sensor array with a 10 s update rate. The measured signal is the change in MR from the initial MR in parts-per-million (ppm).

### Surface functionalization

Sensors were cleaned with sequential addition and removal of 600 µL of acetone, methanol, and isopropanol. The sensor arrays were then placed in an ultraviolet (UV)-Ozone Cleaner (Uvotech Systems, Helios 500) for 10 min. Immediately afterward, a 100 µL solution of 1% Poly(allylamine) in pH 6.0 MES buffer is placed in the sensor wells for 10 min and then washed with 600 µL of deionized (DI) water. The sensors were baked for 90 min at 110°C in a Precision Compact Oven (Thermo Scientific #PR305225G). PEMA is made aqueous by placing it in a 170°C water bath for 90 min directly before adding it to the sensor surface. Then the 100 µL solution of 1.5% aqueous PEMA in pH 6.0 MES buffer is passed through a 0.22 µm filter before addition to the sensors for 5 min. We also tested combinations of 2% PEMA at 200°C and 0.45 μm filters to discover these optimized conditions. The sensors are rinsed with 1 mL DI water, air-dried with an aspirator, and baked for 1 h at 160°C. The protocol was adapted from previous methods (Kim et al., 2013).

### Antibody biotinylation

Sulfo-NHS-LC Biotin with a 22.4-Å spacer (Thermo Scientific, #A39257) was diluted in ultrapure water and added at a 20:1 ratio of label to purified detection antibodies diluted in PBS pH 7.4. The conjugation was incubated for 2 h on ice. Unincorporated biotin was removed using a desalting spin column with a 7 kDa molecular weight cut-off (Thermo Scientific, #89882).

### Antibody spotting

Individual sensors were spotted with capture antibodies using an iTWO-300P automated spotter (axiVEND, Florida). Twenty droplets of ∼100 pL were spotted on each sensor to cover the sensor surface. A printing buffer (1 M Betaine and 12.5% 2,3-Butanediol in PBS) is needed for the IBP4 capture antibodies. IBP4 capture antibody was spotted on each sensor by transferring 100 pL twice from a stock of 0.34 mg/mL. The SHBG capture antibody is spotted in 10% glycol in PBS at 0.125 mg/mL. In general, 16 sensors are spotted with the capture antibodies, while the remaining sensors are spotted with either 1% BSA (a negative control to monitor for nonspecific MNP binding), 0.1 mg/mL IL-6 capture antibody (a negative control for nonspecific antibody interactions), and 1 mg/mL BSA-Biotin (a positive control for biotin-streptavidin interactions), or an amine-PEG-Biotin substrate (another positive control for biotin-streptavidin interactions). After spotting, the automated spotter chamber is brought to 70% humidity for 1 h then the sensors are left to incubate overnight in the chamber.

### Magnetic immunoassay

The sensor array is placed in a Teflon holder with silicon o-rings. Then the sensors are washed with 600 µL of Buffer 1 (0.1% BSA, 0.1% Tween-20 in 1 × PBS) and blocked for 30 min using 5% BSA in PBS. Following an additional wash with 600 µL of Buffer 1, the sample containing antigen is diluted in Buffer 1 and then added to the sensors for 1 h. The sensors are washed 5 × with 600 µL of Buffer 1. Biotinylated detection antibodies are diluted in PBS to 10 μg/mL, and 100 µL is added to the sensors for 1 h. Then the sensors are washed 3 × with 600 µL of Buffer 1 and submerged in 100 µL PBS. The sensor array is placed into the magnetic reading station, and 50 µL of magnetic nanoparticles are added to the reaction well.

### Dual-binding magnetic immunoassay

The assay is similar to the magnetic immunoassay described above, but additional steps are added at the end. After the MNPs have reached binding equilibrium with the detection antibodies (∼30 min), the unbound MNPs are washed away, and 10 mM free biotin is added for 15 min before being washed away with 600 µL of Buffer 1. Then 50 µL of 10 nM SHBG is added and incubated for 40 min with 20 µL of 75 μg/mL detection antibody for the last 20 min. The well is then washed with 600 µL of Buffer 1 before 25 µL of MNPs are added for 20 min for an additional binding curve. The normalization ratio is calculated by dividing the first curve’s saturation value by the second curve’s saturation value on a sensor-by-sensor basis.

### Anti-mouse assay

After antibody spotting and overnight incubation, the surface is blocked with 30 min of 100 μL at 5% BSA. Subsequently, 100 μL at 10 μg/mL of anti-mouse detection antibodies are incubated for 1 h. Then the detection antibodies are washed away before PBS is added. The assay is then run with 50 µL of MNPs, and the change in magnetic resistance is quantified after signal saturation.

### Mass spectrometry (MS) assay

Pools of serum samples were generated to span low to high levels of IBP4 and SHBG, respectively (10 pools total). After pooling the individual samples, aliquots were analyzed according to the standard operating procedure. Briefly, pooled serum (50 µL) was immunodepleted of the top fourteen most abundant plasma proteins (Agilent Technologies, #MARS14). Depleted serum was digested with trypsin, spiked with purified stable isotype standard (SIS) peptides, and desalted. Peptides were separated by reverse-phase liquid chromatography and analyzed by multiple reactions monitoring mass spectrometry, with IBP4 and SHBG measured in each of the ten pools. Two relative peptide amounts were quantified as the response ratio (RR) of the endogenous peak area divided by the SIS peak area. Proteomic scores were calculated as the ln (*RR*_IBP4_/*RR*_SHBG_).

### Depleted serum

Pooled serum (1 mL) from female donors was diluted 1:4 in TBS with 0.05% Lauryl Maltose Neopentyl Glycol and mixed with magnetic polyvinyl alcohol beads coupled with anti-IBP4 antibody (6 mg) and anti-SHBG antibody (10 mg). The diluted serum was rotated with beads for 75 min at room temperature, and the depleted serum was removed. This serum was then subjected to a second depletion by the same procedure, resulting in a protein concentration of 16.5 mg/mL and the removal of ∼95% of the detectable IBP4 and SHBG proteins.

### Statistical analysis

All data shown are the mean values with one median absolute deviation as error bars. The limit of blank (LOB) is calculated as 1.645 × the standard deviation of the blank plus the mean of the blank, whereas the limit of detection (LOD) is defined as the LOB plus 1.645 × the standard deviation of the lowest concentration sample (Armbruster and Pry, 2008). The concentration corresponding to the LOD is calculated using the four-parameter logistic (4-PL) coefficients. 4-PL curve fitting was performed using NumPy (v1.18.5) in Python (v3.8) with least squares optimization on the spiked samples in the calibration curve, and the fit parameters were used to back-calculate the concentration of unknown samples. Statistical analysis (Pearson’s coefficient and Deming analysis) was done with NumPy (v1.18.5) and SciPy (v1.6.0) in Python (v3.8). Receiver operator characteristic (ROC) curves were calculated using scikit-learn (v1.0.2) in Python (v3.8).

## Results and discussion

### Antibody immobilization

Antibody coupling to the sensor surface is critical in an immunoassay. Several immobilization chemistries were explored (*e.g.*, Polyethylenimine, (3-Aminopropyl)triethoxysilane, N-Hydroxysuccinimide with 1-Ethyl-3-(3-dimethylaminopropyl)carbodiimide (NHS-EDC), Diaza-Silane, and PEMA-PAAM), and evaluated based on their stability, reactivity, and coverage (Supplementary Figure S4). PEMA-PAAM consistently performed the best and was used for all subsequent experiments to covalently couple the free amines of the SHBG and IBP4 antibodies to the silicon dioxide surface via the anhydride groups. Unbound anhydride groups were blocked by adding an excess of bovine serum albumin (BSA). The sample (in buffer or serum) containing SHBG and IBP4 was added with a non-ionic surfactant (Tween-20) to facilitate binding to the surface-immobilized antibodies and minimize nonspecific interactions. The analyte was detected by adding a biotinylated SHGB or IBP4 antibody, followed by streptavidin-coated MNPs. The MNPs bind to the detection antibodies via a biotin-streptavidin interaction, leading to a change in the local magnetic field proportional to the analyte concentration. With 80 sensors available on each array, multiplex detection of SHBG and IBP4, in addition to positive and negative controls, is possible.

The sensors were spotted with an automated robotic spotter to reduce the sensor-to-sensor variation due to liquid handling. Each of the 120 × 120 μm^2^ sensors is covered by individual droplets (Supplementary Figures S3B,C), eliminating edge effects (e.g., coffee ring) when manually spotting (which is limited to droplets covering 4-6 sensors). The antibody concentration was kept lower than 1 mg/mL, and a printing buffer was necessary to ensure accuracy and consistency in droplet volume. These buffers prevent the protein from binding to the spotter tip and preserve the reagent in solution on the sensor surface longer, prolonging the amine coupling time. Since the surface chemistry forms a covalent bond between anhydride groups and free amines (found in amino acids like lysine distributed throughout the antibodies), the orientation of the antibody on the surface is unpredictable. Some printing buffers can facilitate the orientation of antibodies, allowing for increased interactions between the epitopes of the antibodies and antigen in solution. The ability to spot individual sensors allows the capture antibody concentration and printing buffer to be optimized using a single sensor array. After a study exploring several printing buffers, it was found that 1 M Betaine and 12.5% 2,3-Butanediol in PBS for IBP4 capture antibodies and 10% ethylene-glycol in PBS for SHBG capture antibodies performed the best (Supplementary Figure S5). The sensor array was spotted with a lower protein concentration multiple times to maintain the surface area of the spotted reagent while increasing the concentration.

### Surface chemistry optimization

**FIGURE 2:**
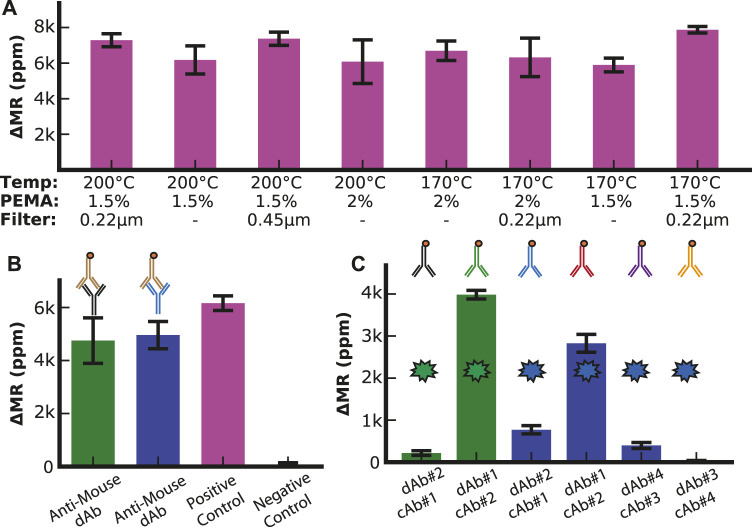
Assay optimization. **(A)** Optimization of surface chemistry parameters with positive control (1 mg/mL biotinylated BSA). **(B)** Anti-Mouse assay (10 μg/mL biotinylated anti-mouse antibody) to analyze the surface density of capture antibodies with positive control (1 mg/mL biotinylated BSA), negative control (1% BSA), SHBG capture antibody (green bar), and IBP4 capture antibody (blue bar). **(C)** Antibody configuration optimization. The two green bars show the signal when the two SHBG antibodies (0.125 mg/mL) are used in both permutations for capture and detection antibodies (dAb). The blue bars show the signal when the two pairs of IBP4 antibodies (0.34 mg/mL) are run in each permutation.

UV-ozone and oxygen plasma treatments have been used to create conditions conducive to coupling antibodies to the surface of the sensor arrays. However, prior experiments have shown that UV-ozone is preferable because oxygen plasma can damage the sensor (Supplementary Figures S6, S7). We optimized other conditions promoting antibody coupling, such as the PEMA concentration, temperature, and filtration pore size (Figure 2A). We designed a simple assay consisting of a linker molecule (PEG with an amine group on one end and biotin on the other) to quantify the change in magnetoresistance (ΔMR) upon binding of streptavidin-coated MNPs. The best response (signal amplitude relative to standard deviation) was for 1.5% PEMA, 170°C, and a 0.22 µm filter. These conditions were used for all subsequent assays.

To quantify the loading of the mouse capture antibodies to the sensor surface, we utilized biotinylated anti-mouse antibodies that directly bind to the capture antibodies and compared the binding to a positive control sensor that was functionalized with biotinylated BSA (Figure 2B). Based on proximity-based detection, GMR sensors are primarily influenced by magnetic nanoparticles close to the sensor surface (Osterfeld et al., 2008). Antibodies (∼8.4—13.7 nm, depending on the orientation) are larger than biotinylated BSA (∼7 nm); thus, we expected that the MNPs bound by the antibodies would be further away from the sensor surface than in the BSA assay, resulting in a lower signal (Tan et al., 2008; Yohannes et al., 2010). We found that the signal for each antibody was between 4.7 k and 5 k ppm, while the biotinylated BSA was 6.2 k ppm. These measurements demonstrated that the capture antibodies were anchored to the surface with sufficient density.

Next, we optimized the antibody orientation (*i.e.*, capture vs. detection and pairing) from the limited antibodies available. Immunoassays were run using all shown paired permutations of capture and detection antibodies with a fixed target analyte concentration of 10 nM in PBS with 0.1% BSA as a carrier protein. With anti-SHBG#1 as the capture antibody (cAb#1) and anti-SHBG#2 as the detection antibody (dAb#2), we measured a signal of 210 ± 50 ppm (Figure 2C). However, when these antibodies were set up in the reverse orientation (cAb#2 with dAb#1), the signal increased by 18.5-fold to 3.9 k ± 100 ppm, demonstrating the importance of this optimization experiment. The same experiment was performed for IBP4, where we found that anti-IBP4#4 as the capture antibody (cAb#4) and anti-IBP4#3 as the detection antibody (dAb#3) had the lowest signal (30 ± 10 ppm) while anti-IBP4#2 as the capture antibody (cAb#2) and anti-IBP4#1 as the detection antibody (dAb#1) had the highest signal of 2.8 k ± 200 ppm. We used these antibody combinations for all subsequent assays. While increasing the concentration of the detection antibodies above 20 μg/mL led to a small signal increase, the increase was not worth the cost of using twice the amount of detection antibodies per assay, so the detection antibody concentration was set at 10 μg/mL (Supplementary Figure S8). In addition, the number of times the SHBG antibodies are run through the biotinylation protocol led to changes in signal when the reagents were bound directly to the sensor surface, indicating that excess biotinylation prevents functionalization of the antibodies to the sensor surface, while insufficient biotinylation led to a decrease in MNP binding (Supplementary Figure S9).

### IBP4 assay

**FIGURE 3:**
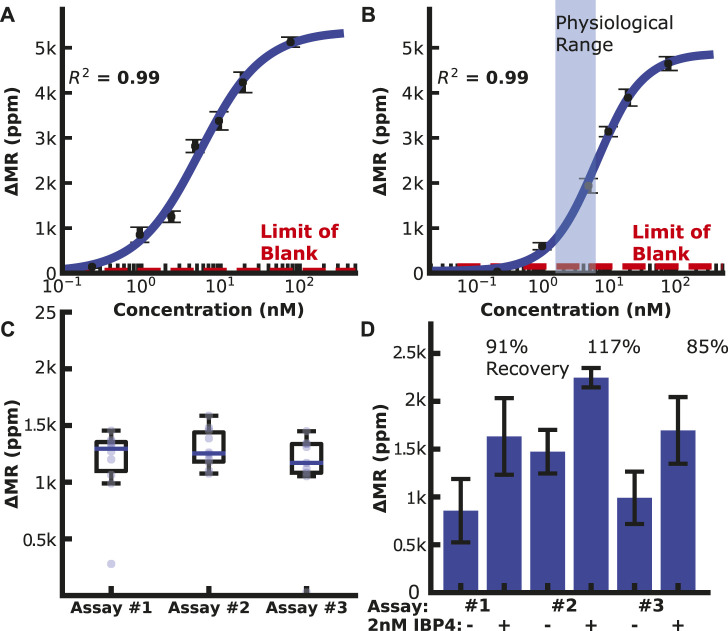
IBP4 assay data. **(A)** IBP4 spiked in PBS (LOD ∼120 pM). **(B)** IBP4 spiked in 1:10 depleted serum (LOD ∼150 pM). **(C)** Reproducibility of the 2.5 nM IBP4 assay in PBS. **(D)** Spike and recovery of 2 nM IBP4 in pooled serum.

We then generated calibration curves by diluting IBP4 into PBS or spiking it into pooled human serum from pregnant donors depleted of endogenous IBP4, as shown in Figures 3A,B. The assay performed well in both sample matrices covering the physiological range (10—60 nM) with a limit of detection (LOD) of 119 and 148 pM in PBS and serum, respectively. Prior experiments showed that a 1:10 dilution is needed to lower the serum’s matrix effect on the anti-IBP4 antibodies (Supplementary Figure S10). Without this dilution, the IBP4 assays were inconsistent. An assay with 2.5 nM IBP4 spiked in Buffer 1 was run multiple times (10 replicates with 3 sensor arrays) to assess the assay-to-assay variability. This concentration was selected considering the linear range of the assay. The average signal was 1.2 k ppm, and the coefficient of variation was 7% (Figure 3C). For comparison, the average of the negative control sensors (non-complementary antibody or BSA) was 60 ± 5 ppm. We further quantified the accuracy of the assay through spike and recovery studies. Blinded serum pools were measured, with some having 2 nM IBP4 spiked into the sample. The concentrations were back-calculated using the serum calibration curve. The assays accurately quantified the spiked-in analyte within 15% (Figure 3D). Collectively, these data demonstrate the performance of the IBP4 assay showing repeatable, accurate detection in serum.

### SHBG dual-binding magnetic immuno assay

**FIGURE 4:**
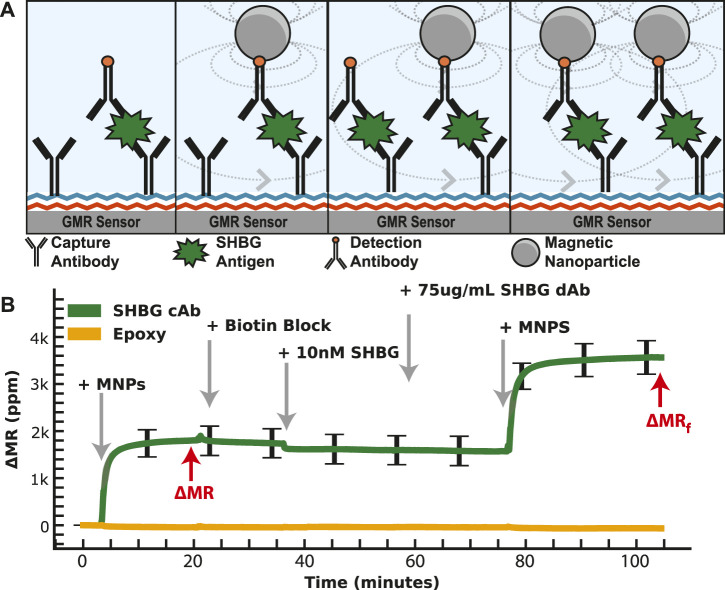
Dual-binding magnetic immunoassay. **(A)** Illustration of the dual-binding assay where a signal is first generated from the antigen in the sample. After binding has saturated, a known value of the calibrant is assayed on top of the existing assay. **(B)** Measured signal time course showing the binding curves from the dual-binding SHBG assay. The first binding curve is from 1 nM SHBG, then 10 nM SHBG is added. The ratio of the first to the second saturated value is used to normalize away assay variation.

We then generated a concentration curve with SHBG diluted into PBS with a LOD of 21 pM. A similar study as IBP4 was performed for the SHBG assay; however, the reproducibility was highly variable, with a coefficient of variation >17%. Many attempts to improve this using different antibody pairings and surface chemistries yielded mixed results but did not solve the underlying issue. There are several possible reasons for this variability, such as inconsistent surface antibody orientation and/or analyte dimerization/aggregation that may complicate immunoassay results in some formats despite robust SHBG measurement by mass spectrometry and by clinical analyzers (Grishkovskaya et al., 2000; Bradford et al., 2017). Since the number of affinity reagents selected by our collaborator for this analyte was limited, we could not simply swap out affinity reagents; instead, we devised a way to deal with the variation at the assay level. Specifically, we modified the classical immunoassay by introducing another binding step with a known concentration. As depicted in Figure 4, the first phase of the assay proceeds identically to the standard magnetic immunoassay with the functionalization of capture antibodies. After the first binding phase, we remove the unbound MNPs and add free biotin to block all unbound streptavidin on the tethered MNPs. This step is necessary to preserve the one-to-one relationship between the analyte and the MNPs (and thus the signal generated) and not deplete the biotinylated detection antibodies subsequently added. Next, we add a known concentration of SHBG protein on top of the already-bound protein. This complex is incubated for 20 min, followed by adding more biotinylated detection antibodies and MNPs. This “dual-binding” assay allows the signal from the first binding event to be normalized to the second binding event with a known concentration, resulting in a ratiometric signal. The important thing to note is that if there is inhomogeneity in the density of accessible capture antibody (due to surface chemistry, antibody orientation, etc.), it is also there for the second binding event and normalized out when taking the ratio. If it was a global effect that affected *all* antibodies similarly, one could normalize the signal to a housekeeping protein or an orthogonal spiked protein; however, this was not our situation. The issue was particular to the SHBG capture antibody, necessitating a different approach. The choice of 10 nM (vs. another concentration) spiked protein was based on the desire to operate in the linear region of the calibration curve—thus, any amount spiked on top of the sample required us to have sufficient dynamic range. The increase in signal after 10 nM SHBG incubation is correlated with the number of available antigen-binding sites after the sample incubation period, which means that the sample quantitation is less dependent on having the same number of initial antigen-binding sites on every sensor and across different assays. We also explored spiking in SHBG at a concentration that saturated the sensor, but this required significantly more reagents. While the process is more complex than the magnetic immunoassay, we intend to automate it using a microfluidic cartridge such that the operator simply adds the sample.

**FIGURE 5:**
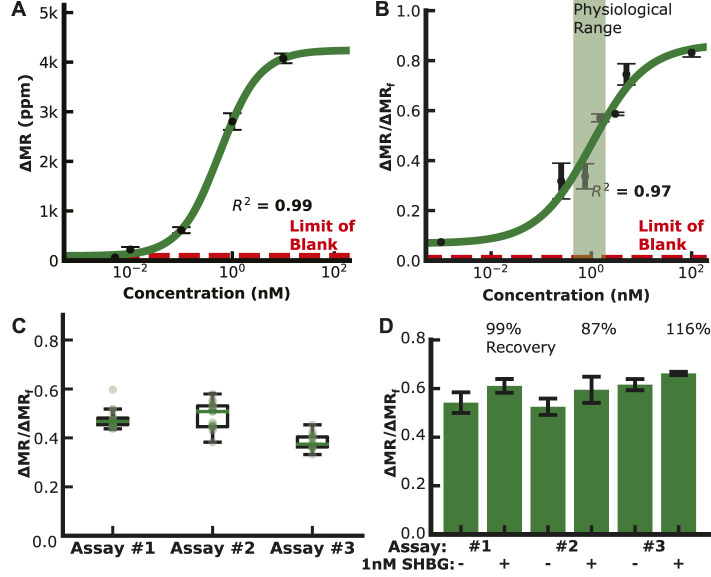
SHBG assay data. **(A)** Calibration curve in PBS (LOD ∼20 pM). **(B)** Calibration curve in depleted serum (LOD ∼15 pM). **(C)** Reproducibility assays with 1 nM SHBG in buffer. **(D)** Spike and recovery assays with 1 nM SHBG in pooled serum.

We measured SHBG in PBS and diluted serum using the dual-binding assay to generate a calibration curve (Figures 5A,B). The endogenous level of SHBG is high (100 nM), requiring a higher dilution of 1:1000 to bring it within the assay’s dynamic range. The SHBG assay covered the physiological range of 100 nM for non-pregnant women, 600 nM in the first trimester, 1,000 nM in the second trimester, and 1,200 nM at delivery (Ekelund and Laurell, 1994) with a 13 pM LOD. The assay reproducibility is shown in Figure 5C, where the coefficient of variation for multiple 1 nM SHBG dual-binding assays is 10%—a significant improvement over the classical magnetic immunoassay with a coefficient of variation of over 17%. Spike and recovery assays using the dual-binding assay with 1 nM SHBG in pooled serum had a quantitation accuracy within 15% (Figure 5D). Nonspecific binding was measured by running an assay with serum depleted of SHBG and IBP4. Sensors with capture antibodies for SHBG and IBP4 developed little signal despite the addition of detection antibodies, which signifies that the antibodies are specific for SHBG and IBP4 (Supplementary Figure S11). The SHBG capture antibodies developed 56 ± 14 ppm signal, while the IBP4 capture antibodies developed 51 ± 14 ppm of signal, similar to the 28 ± 5 ppm of signal from the negative control. These data demonstrate that the SHGB dual-binding assay can reproducibly and quantitatively detect the target analyte over the physiological range throughout gestation.

### Cross-reactivity

Despite running the previous assays in serum, we wanted to ensure that the assay was specific and had no cross-reactivity since quantitation is important for the proteomic score. The assay cross-reactivity was evaluated by adding 10 nM of SHBG to an IBP4 assay and 10 nM of IBP4 to an SHBG assay. The sensors spotted with off-target capture antibodies exhibited a negligible signal, no more than 120 ppm, similar to the negative control (BSA and IL-6) sensors (Supplementary Figure S12). Assays with serum immunodepleted of IBP4 and SHBG exhibited similarly low signals (Supplementary Figure S11). These data demonstrate that the assay is highly specific to the target proteins with no detectable cross-reactivity.

### Assay cross-validation

**FIGURE 6:**
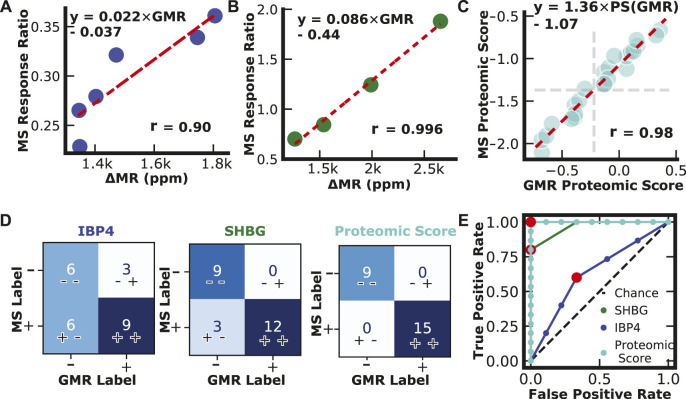
Assay Validation. **(A)** IBP4 GMR values from pooled serum plotted against IBP4 response ratio values from mass spectrometry. **(B)** SHBG GMR values from pooled serum plotted against SHBG response ratio values from mass spectrometry. **(C)** Proteomic scores calculated from measurements of pooled serum combinations (*n* = 24) using the reported GMR assay and mass spectrometry. Proteomic scores are calculated by taking the natural log of the SHBG and IBP4 ratio. **(D)** Confusion matrices showing classification results from single biomarkers and proteomic scores. **(E)** Receiver operator curves for IBP4, SHBG, and proteomic score.

Serum patient pools were created and provided to the researchers blinded by collaborators at Sera Prognostics. The pools covered most of the physiological range of IBP4 (16—40 nM) and SHBG (567—1247 nM) throughout gestation. Each sample was run independently using the reported magnetic immunoassays and the clinically, analytically validated mass spectrometry assays. There was a strong correlation between the two different assays for IBP4 (*n* = 6) and SHBG (*n* = 4), as shown in Figures 6A,B. A proteomic score paired with clinical factors was shown to accurately predict spontaneous preterm birth, where the proteomic score is defined as the natural log of the IBP4 divided by SHBG response ratios (Saade et al., 2016). Because IBP4 and SHBG were not measured from the same pooled sample, to demonstrate the concordance of the proteomic score between the assays, IBP4 values from the pools selected for their span of IBP4 concentrations (data in Figure 6A) were ratioed against pools selected for their SHBG concentrations (data in Figure 6B) with all possible permutations calculated (*i.e.*, for each IBP4 value, a ratio was made using 4 different SHBG values, for a total of 24 combinations, as shown in Supplementary Table S3). The scores were calculated for both assays and plotted against each other. Figure 6C shows that the assays exhibit high similarity with a Pearson correlation coefficient of 0.98. The proteomic score threshold value for the mass spec assay is −1.37, which translates to a magnetic immunoassay score of −0.22. We then assessed the concordance of the two assays for each biomarker independently, and the scores derived from the ratio of the two using the mass spectrometry data as the “true” outcome. As shown in Figure 6D, using SHBG or IBP4 alone has lower concordance between the two assays. When using the proteomic score, the reported assay has 100% positive and negative agreement. Finally, we calculated the ROC curve for the three cases to optimize the threshold value (Figure 6E). A −0.3 cut-off value for the proteomic score resulted in the best performance with an area under the curve (AUC) of 1, similar to the value predicted from the correlation analysis in Figure 6C. The sample size here was limited but perfectly agreed with the clinically validated mass spectrometry-based assay.

### Point of care testing

A magnetic immunoassay enables the possibility of POCT, greatly expanding accessibility and reducing the turnaround time. Since the sensor arrays have 80 sensors, there is room to add more biomarkers once identified. Microfluidics would allow for automation to reduce the expertise needed to run the assay and facilitate multiple sample dilutions to be run on the same chip. In addition, automated dilutions for logarithmic concentrations by microfluidics have been demonstrated and could be implemented for ease of use in a point-of-care setting and to allow a sample to be diluted 1:10 for IBP4 and 1:1000 for SHBG on a single sensor array (Kim et al., 2008). Paired with microfluidics, which can be developed in future work, these sensors could eventually lead to a comprehensive pregnancy panel, allowing ultra-personalized care.

Future work has various avenues for expediting the assay time. Promising strategies include active attraction of MNPs and wash-free assays (Choi et al., 2016; Su et al., 2019). While these approaches hold potential, it is worth highlighting that the present emphasis rests on establishing the fundamental viability of the assay concept. Should improving the assay’s efficiency encounter challenges in significantly curtailing the assay time, one solution might involve incorporating a brief waiting period preceding an obstetrician appointment, which ensures that healthcare providers can access the assay results in tandem with the patient’s visit.

Another significant advantage of the reported assay is the low sample volume. All assays were run with less than 50 µL of sample, allowing them to be run from a single finger-puncture procedure (Serafin et al., 2020). This volume remains the same as more targets are added to the assay panel. The blood from a finger-puncture procedure could be diluted with microfluidics, and the assay steps automated. The clinical application of a resulting device will require full analytical and clinical validation. Clinical validity must demonstrate discrimination and calibration for the association of the derived multi-analyte score with the risk of sPTB (Alba et al., 2017). The expansion of GMR sensor technology allows for personalized health monitoring of fetal development, and further automation will allow the test to be accessible to many pregnancies.

## Conclusion

This work reports a protein biomarker assay for predicting spontaneous preterm birth. Quantitative assays were developed and optimized for SHBG and IBP4 in maternal serum. The assays exhibited high sensitivity (pM-level LOD) with a relatively broad dynamic range that covers the physiological level of the analytes as they change throughout gestation. Based on the limited affinity reagents available, a novel dual-binding assay was developed to address underlying variability in the SHBG assay enabling self-normalization of the response—significantly improving the repeatability and accuracy. Concordance of the GMR assays was then demonstrated by measuring pooled serum samples covering the entire target analyte range and compared against the mass spectrometry assays. The GMR and central laboratory MS assay had a calculated analytic agreement of 100%. This report demonstrates a step toward the clinical application of our device after full analytical and clinical validation. Additionally, future work will center on automating this assay in a microfluidic cartridge format to eliminate currently required assay expertise, adding other validated preterm birth biomarkers, and adding other pregnancy-related biomarkers, including miRNA targets, to improve fetal and maternal health outcomes.

## Data Availability

The original contributions presented in the study are included in the article/Supplementary Material, further inquiries can be directed to the corresponding author.
