# Supplementary material for: A dual-binding magnetic immunoassay to predict spontaneous preterm birth
Source: Front Bioeng Biotechnol. 2023 Sep 13;11:1256267. doi: 10.3389/fbioe.2023.1256267 (PMC10542577; doi:10.3389/fbioe.2023.1256267)
Supplement: Supplementary file 1 [file DataSheet1.pdf]

A Dual-Binding Magnetic Immunoassay to Predict Spontaneous Preterm Birth

Supplementary Material

1 Supplementary Figures and Tables

1.1 Supplementary Figures

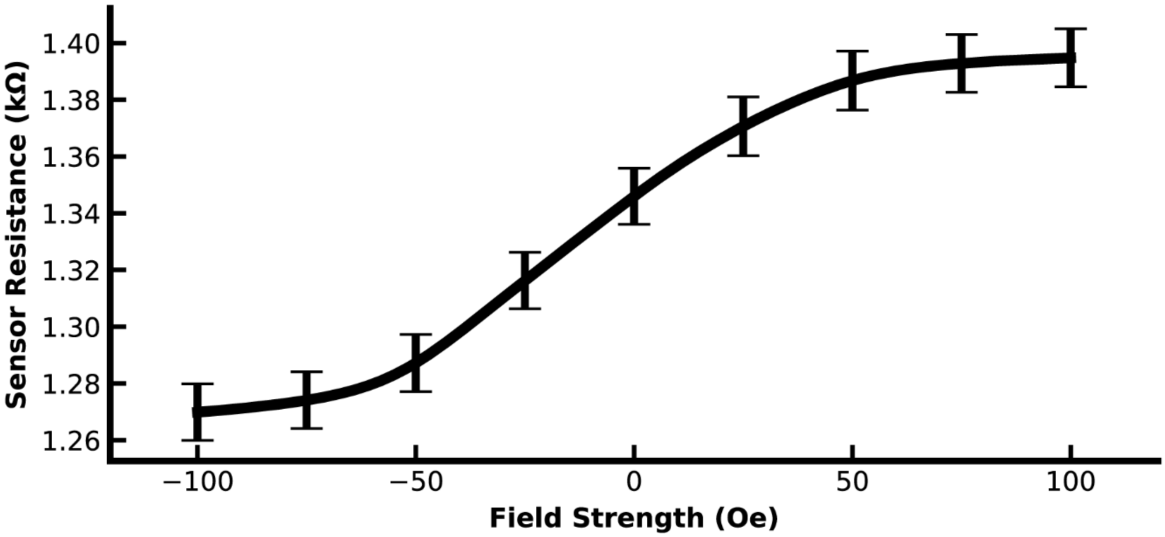

Supplemental Figure 1. GMR SV Transfer Curve. Measured resistance versus magnetic field applied for GMR SV sensor.

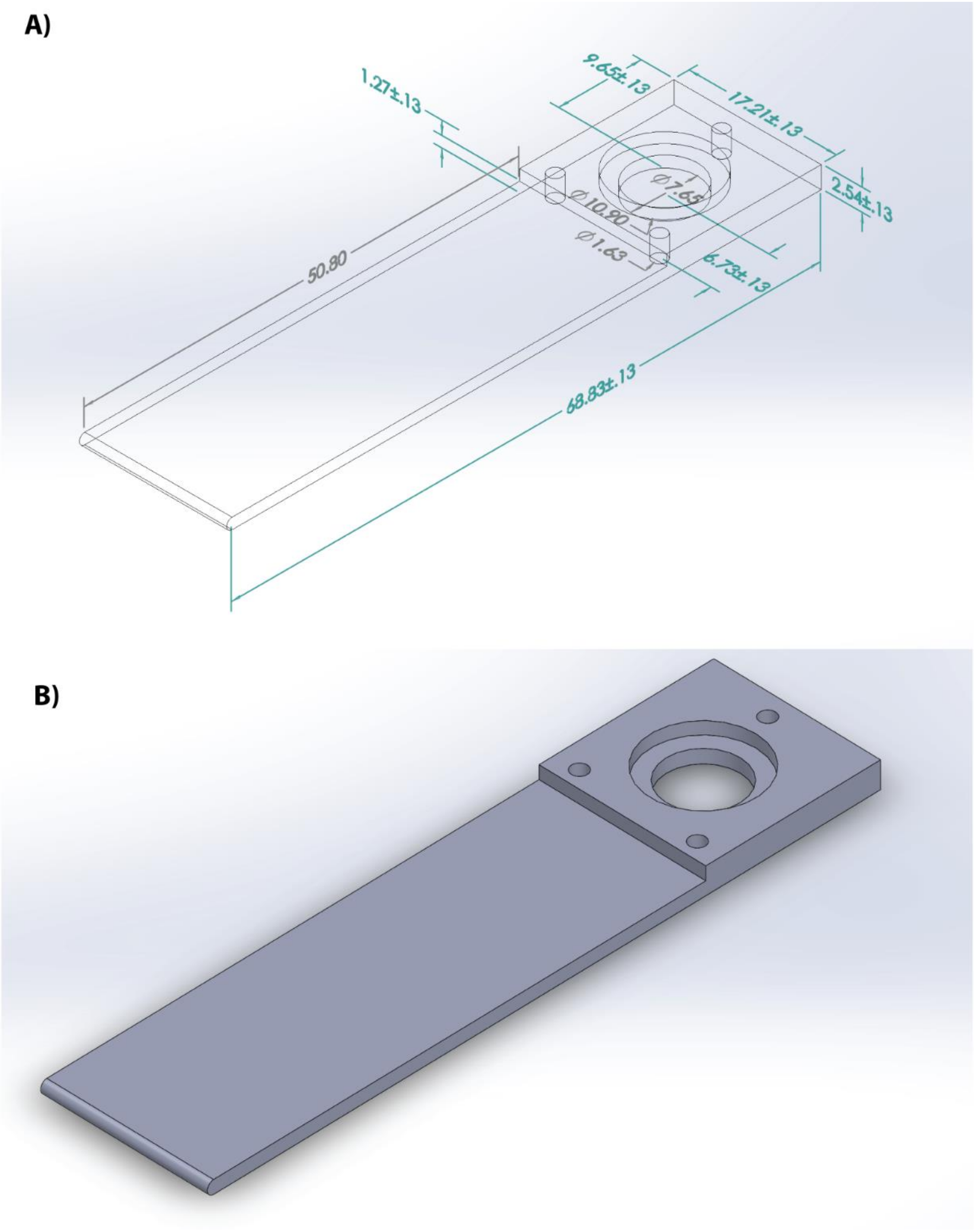

Supplemental Figure 2. Sensor Holder. A) Drawing of Teflon holder used to create a well over the GMR SV sensor array with dimensions in millimeters. **B)** 3D rendering of Teflon holder for GMR SV array.

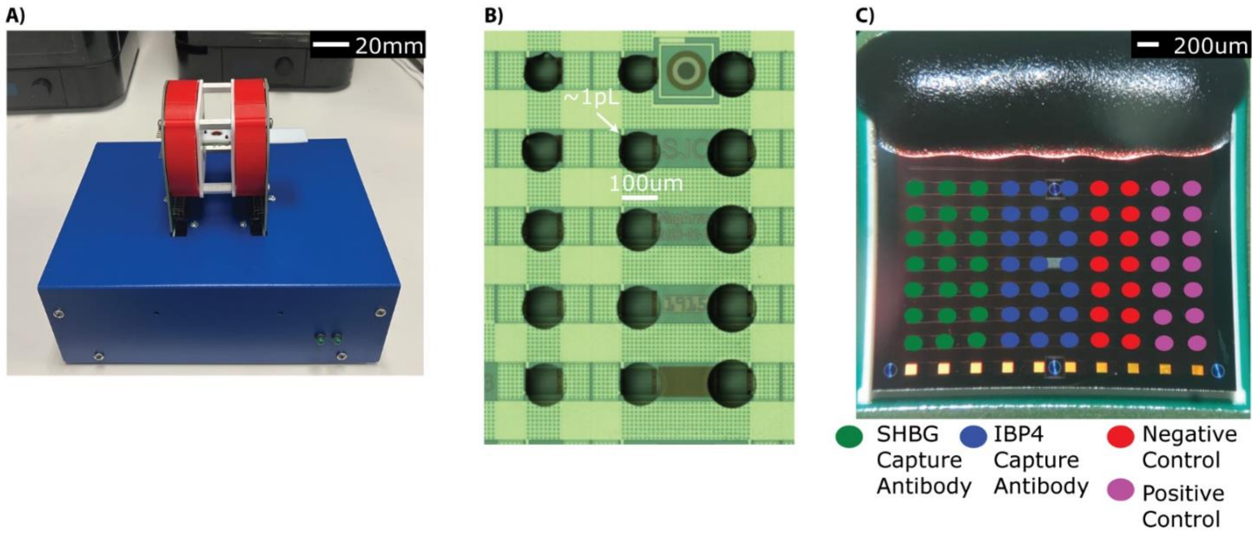

Supplemental Figure 3. GMR reader. A) Photograph of GMR SV reader station with a sensor inside the Helmholtz coil. **B)** Photograph of the GMR SV sensor array with sensors spotted with reagents. **C)** Photograph of the GMR SV sensor array with 80 sensors arranged in an 8×10 matrix where each sensor is 120×120 μm^2 on a 280 μm pitch with an example map of spotting.

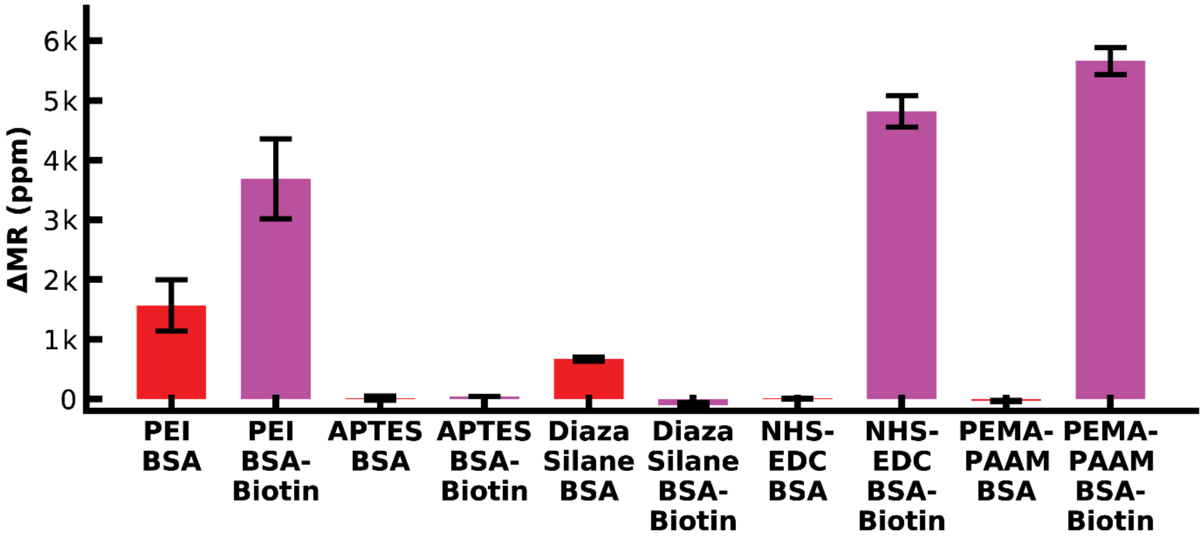

Supplemental Figure 4. Surface Chemistry Alternatives. Positive (red) and negative (magenta) control values for surface chemistries explored.

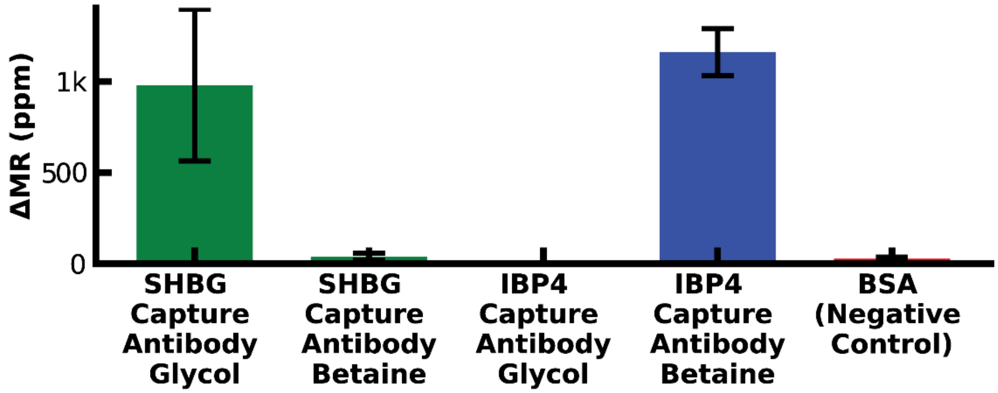

Supplemental Figure 5. SHBG and IBP4 Capture Antibodies in Printing Buffers. 2 nM SHBG assay and 2.5 nM IBP4 assay with glycol printing buffer and betaine printing buffer.

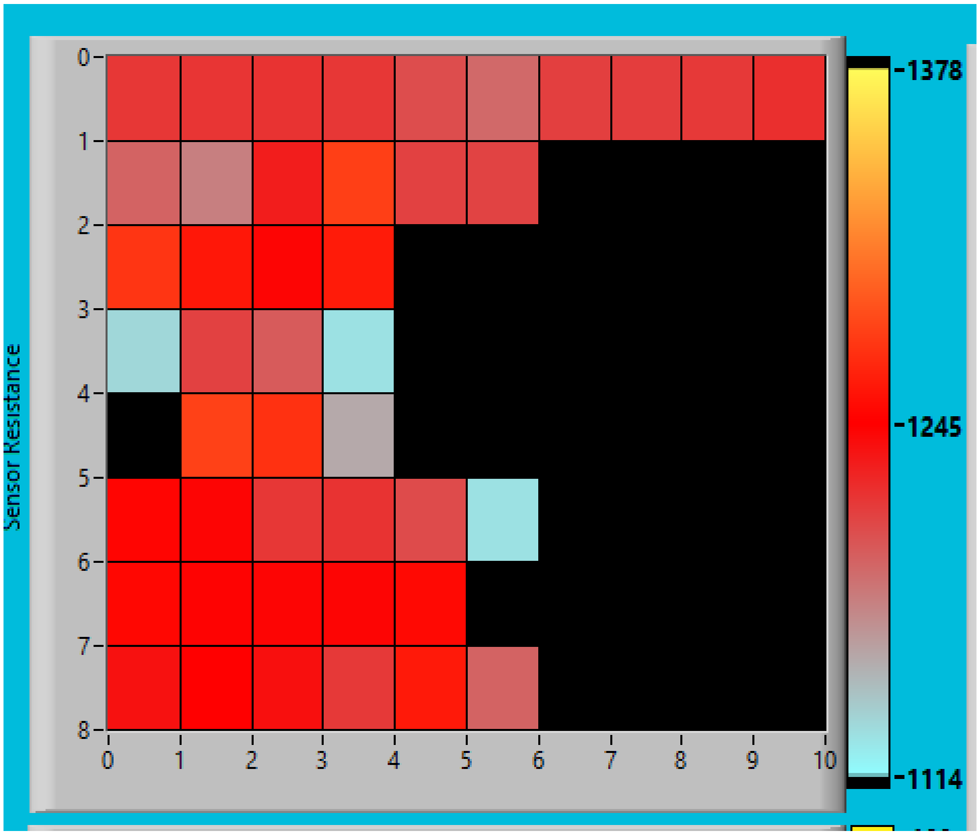

Supplemental Figure 6. GMR Damage from Oxygen Plasma. Image of GMR resistance readings with nearly half the sensors out of range (indicated in black).

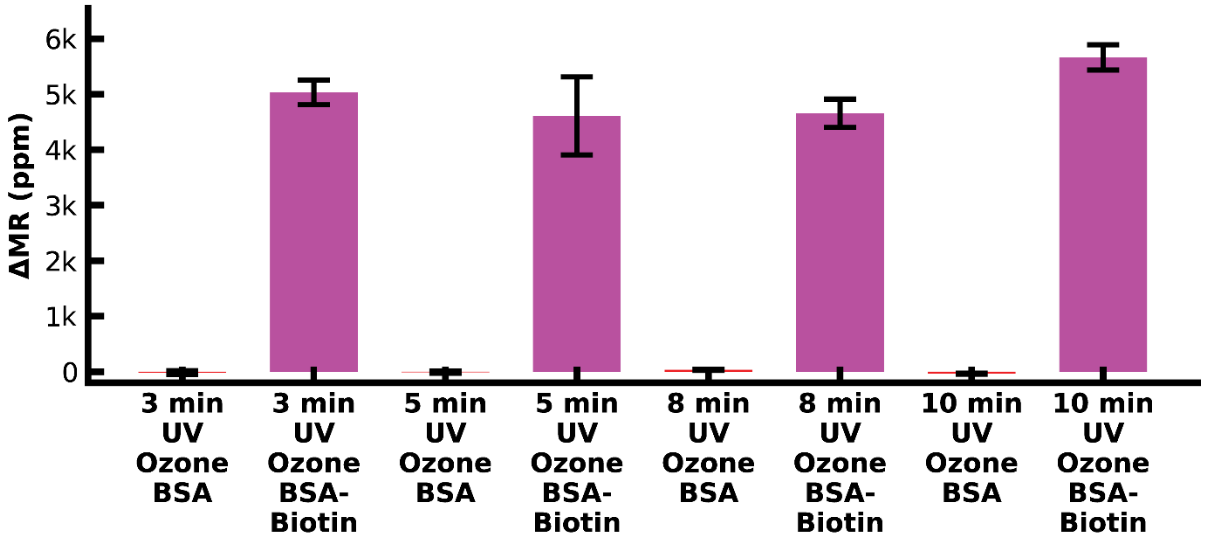

Supplemental Figure 7. UV Ozone Times for PEMA-PAAM Surface Chemistry. Positive (red) and negative (magenta) control values for UV Ozone times explored.

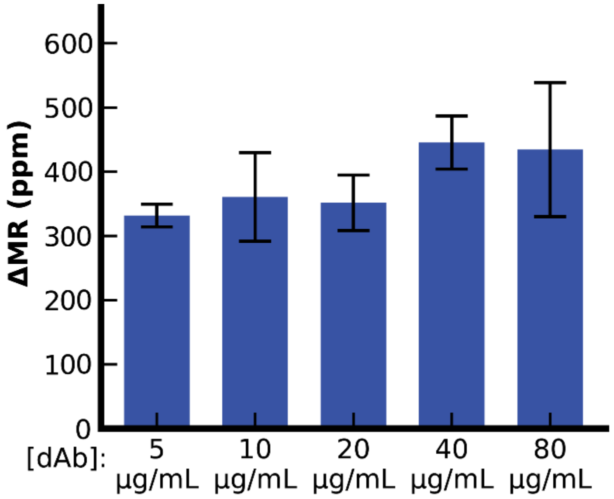

Supplemental Figure 8. IBP4 Detection Antibody Concentration Optimization. Titration of the detection antibody from 80ug/ml to 5ug/mL with 1nM of antigen added.

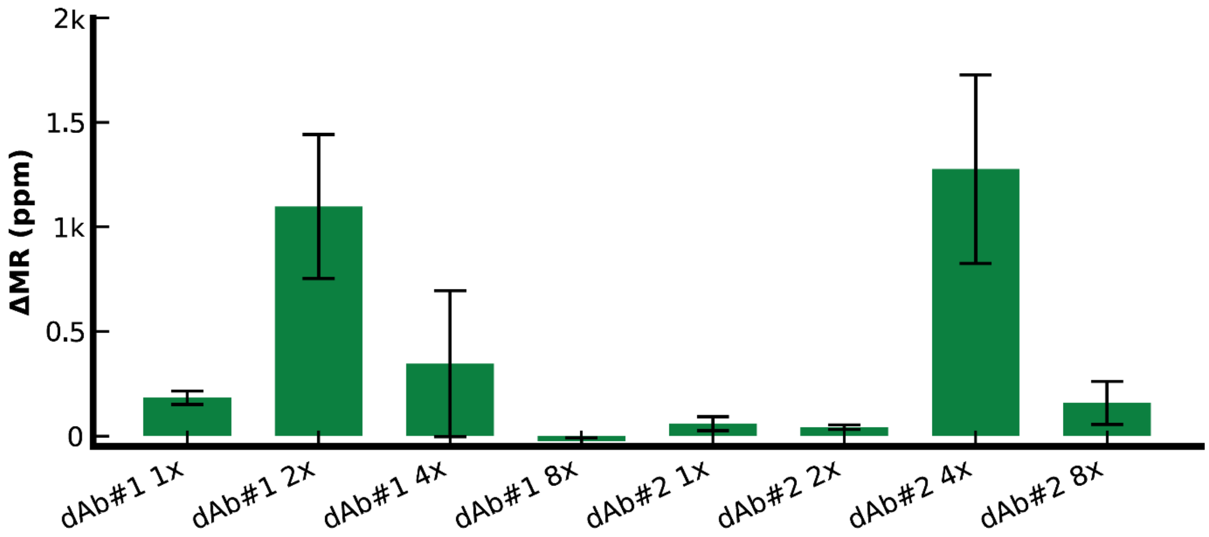

Supplemental Figure 9. Optimization of rounds of biotinylation for both SHBG antibody #1 and antibody #2. These assays show signal from the direct functionalization of the SHBG detection antibodies.

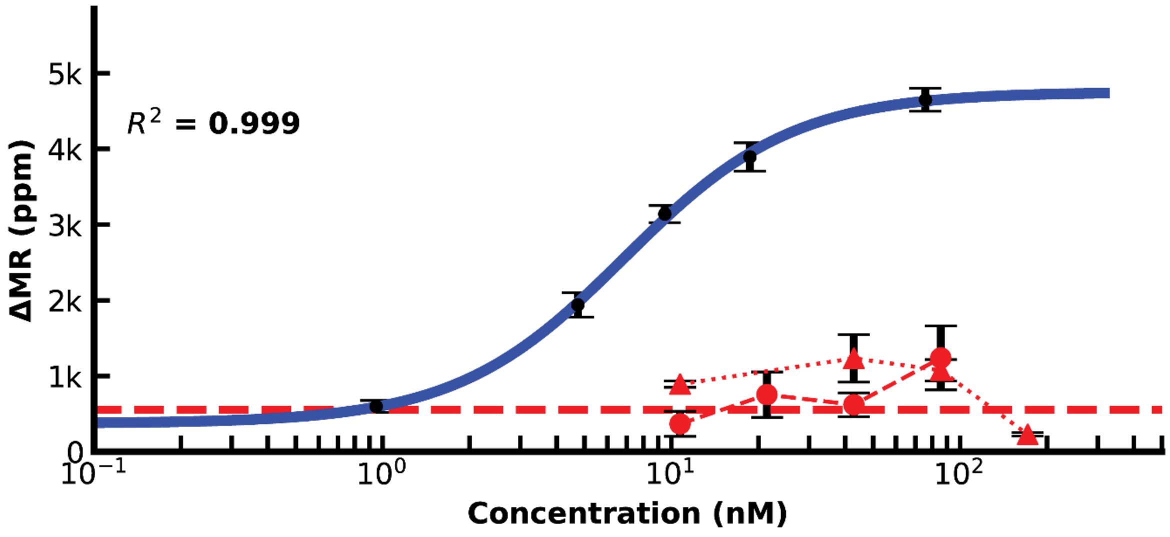

Supplemental Figure 10. IBP4 at Different Serum Dilutions. The blue line is 1/10 dilution, neat serum is the dashed red line, and 1/4 serum dilution is the dotted red line.

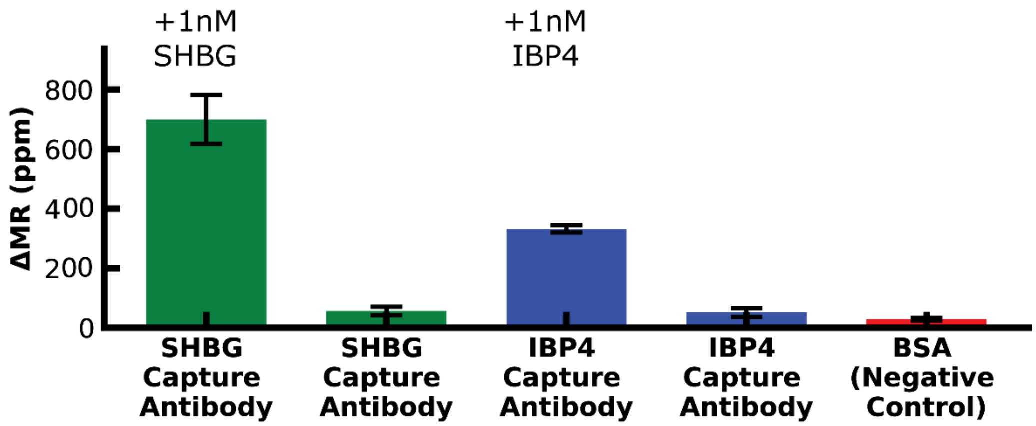

Supplemental Figure 11. Nonspecific Signal. Nonspecific binding SHBG and IBP4 assays in non-spiked depleted serum and 1nM analyte comparison.

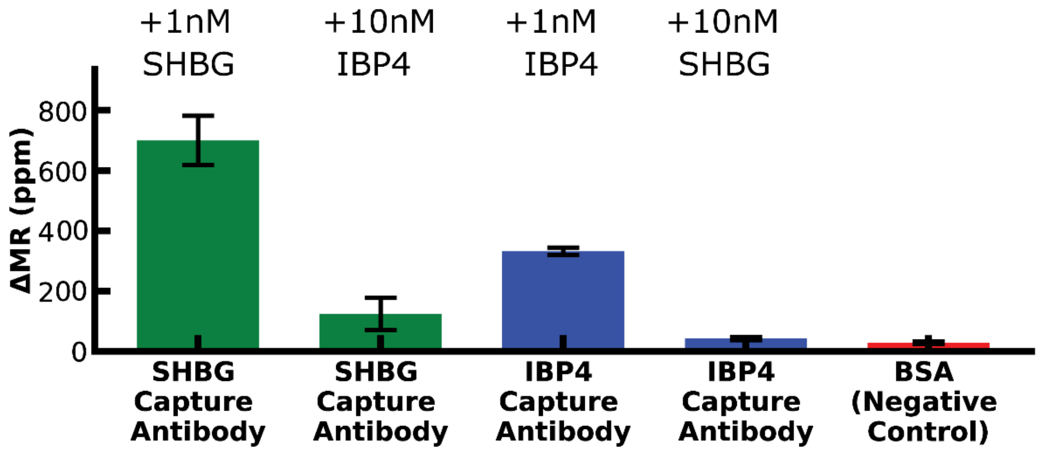

Supplemental Figure 12. Cross-Reactivity. Binding of SHBG and IBP4 assays with 10 nM of the opposite analyte; 1nM target analyte assays for comparison.

1.2 Supplementary Tables

Supplemental Table 1. Antibody properties.

	Host	K_a [$M^{-1}s^{-1}$]	K_d [s^{-1}]	K_D [nM]
IBP4 Capture #2	Mouse	9.03E+04	9.21E-04	10.2
IBP4 Detection #1	Mouse	3.80E+04	6.00E-04	15
SHBG Capture #1	Mouse	6.35E+04	1.50E-04	2.36
SHBG Detection #2	Mouse	4.54E+05	1.50E-04	0.33

Supplemental Table 2. Four-parameter logistic curve fitting parameters.

$$f(x) = \frac{a - d}{1 + \left(\frac{x}{c}\right)^b} + d$$

	a	*b*	*c*	*d*
IBP4 in Buffer	0.88	1.09	5.45	230
IBP4 in Serum	2.93	1.23	6.34	209
SHBG in Buffer	181	-1.11	0.57	3.99
SHBG in Serum	0.87	-0.80	1.02	0.069

Supplemental Table 3. Data used for cross-validation of GMR and mass spectrometry (MS) assay.

Pair	IBP4 (GMR)	SHBG (GMR)	IBP4 (MS)	SHBG (MS)	RiskScore (GMR)	RiskScore (MS)
1	57.634	53.96	0.228475	0.70125	0.06587	-1.12144
2	57.634	65.72	0.228475	0.84485	-0.13129	-1.30773
3	57.634	85	0.228475	1.24355	-0.38854	-1.6943
4	57.634	113.44	0.228475	1.87995	-0.67716	-2.10757
5	57.53	53.96	0.265175	0.70125	0.064064	-0.97247
6	57.53	65.72	0.265175	0.84485	-0.1331	-1.15877
7	57.53	85	0.265175	1.24355	-0.39034	-1.54534
8	57.53	113.44	0.265175	1.87995	-0.67897	-1.95861
9	59.97	53.96	0.27925	0.70125	0.105601	-0.92076
10	59.97	65.72	0.27925	0.84485	-0.09156	-1.10705
11	59.97	85	0.27925	1.24355	-0.34881	-1.49362
12	59.97	113.44	0.27925	1.87995	-0.63743	-1.90689
13	77.153	53.96	0.361	0.70125	0.357547	-0.66399
14	77.153	65.72	0.361	0.84485	0.160387	-0.85028
15	77.153	85	0.361	1.24355	-0.09686	-1.23685
16	77.153	113.44	0.361	1.87995	-0.38548	-1.65012
17	74.62	53.96	0.339	0.70125	0.324166	-0.72686
18	74.62	65.72	0.339	0.84485	0.127005	-0.91316
19	74.62	85	0.339	1.24355	-0.13024	-1.29973
20	74.62	113.44	0.339	1.87995	-0.41887	-1.713
21	62.9	53.96	0.3213	0.70125	0.153303	-0.78049
22	62.9	65.72	0.3213	0.84485	-0.04386	-0.96678
23	62.9	85	0.3213	1.24355	-0.30111	-1.35335
24	62.9	113.44	0.3213	1.87995	-0.58973	-1.76663
